# Biomimetic Approaches to the Synthesis of Natural Disesquiterpenoids: An Update

**DOI:** 10.3390/plants10040677

**Published:** 2021-04-01

**Authors:** Diego Caprioglio, Stefano Salamone, Federica Pollastro, Alberto Minassi

**Affiliations:** 1 Department of Pharmaceutical Sciences, University of Piemonte Orientale, L.go Donegani 2/3, 28100 Novara, Italy; diego.caprioglio@uniupo.it (D.C.); salamone.ste@gmail.com (S.S.); federica.pollastro@uniupo.it (F.P.); 2 PlantaChem srls, via Canobio 4/6, 28100 Novara, Italy

**Keywords:** natural compounds, secondary metabolites, disesquiterpenoids, bioactive compounds

## Abstract

Natural disesquiterpenoids represent a small group of secondary metabolites characterized by complex molecular scaffolds and interesting pharmacological profiles. In the last decade, more than 400 new disesquiterpenoids have been discovered and fully characterized, pointing out once more the “*magic touch*” of nature in the design of new compounds. The perfect blend of complex and unique architectures and biological activity has made sesquiterpene dimers an attractive and challenging synthetic target, inspiring organic chemists to find new and biomimetic approaches to replicate the efficiency and the selectivity of natural processes under laboratory conditions. In this work, we present a review covering the literature from 2010 to 2020 reporting all the efforts made in the total synthesis of complex natural disesquiterpenoids.

## 1. Introduction

Natural products have always been an invaluable source of bioactive compounds targeting important biological endpoints and are continuing to serve as curative agents against different pathologies [1,2]. Their ability to perturbate different molecular targets is probably the result of the *“natural evolution*” occurred in living organisms in order to obtain “*functional molecules*” with antagonistic, antifeedant purposes [3] and in mutual interaction with the environment [4,5]. Secondary metabolites are characterized by a high structural complexity deriving from the work of complex enzymatic systems capable of synthesizing not only monomeric compounds, but also dimeric, trimeric and polymeric frameworks leading to a wide range of interactions with a large number of functional proteins marked by homo- and hetero-oligomeric structures. 

Disesquiterpenoids, or sesquiterpene dimers, are a group of active molecules containing at least 30 carbons, with a high structural variance deriving from homo- or heterodimeric coupling of two sesquiterpenoids units. They are classified in three major classes depending on their biogenetic pathways and their structural features, namely *true disesquiterpenoids*, *pseudo-disesquiterpenoids* and *di-merosesquiterpenoids* [6]. The compounds belonging to the *true disesquiterpenoids* originate from farnesyl diphosphate, with the two sesquiterpenoids moieties linked at list by one C-C bond derived from cycloaddition or free-radical coupling reactions. The *pseudo-disesquiterpenoids* share the same biogenetic pathway of the previous group, but their structures are characterized by an ester, ether or other groups acting as “*connectors*” between the two sesquiterpenes subunits. The last group of dimers derives from hybrid biogenetic pathways leading chimeric structures with the terpenoid moiety linked by a C-C bond with other subunits belonging to different classes of secondary metabolites such as polyphenols and alkaloids.

In the last decade, more than 400 new disesquiterpenoids [7] have been discovered and fully characterized, underling once more the “*magic touch*” of nature in the design of new compounds. The intriguing architectures and the interesting pharmacological profile of sesquiterpene dimers attracted the attention of synthetic chemists in the attempt to duplicate the efficiency and the selectivity of natural processes under laboratory conditions. 

Considering the growing interest around this group of secondary metabolites, the final aim of this review is to cover the literature between 2010 and 2020 relatively to the efforts made in the total synthesis of natural disesquiterpenoids, emphasizing not only the synthetic strategy but also the pharmacological profile of the molecules analyzed. The compounds will be grouped according to the class of the terpenoids subunits.

## 2. Bisabolane Dimers

### 2.1. Meiogynin A

Meiogynin A (**1**) is a bisabolane dimer possessing an original *cis*-decalin skeleton with five stereocenters and a carboxylic moiety at the ring junction. Isolated from the bark of *Meiogyne cylindrocarpa* in 2009 [8], it displays a strong inhibition activity against the anti-apoptotic protein Bcl-xL. The latter is a member of the Bcl-2 family of proteins playing a crucial role in the regulation of the programmed cell death in several eukaryotic systems. Bcl-xL has emerged as in interesting anti-cancer target due to its overexpression in prostate cancer cells and its role in establishing the multidrug resistance phenotype [9,10,11]. 

**Scheme 1:**
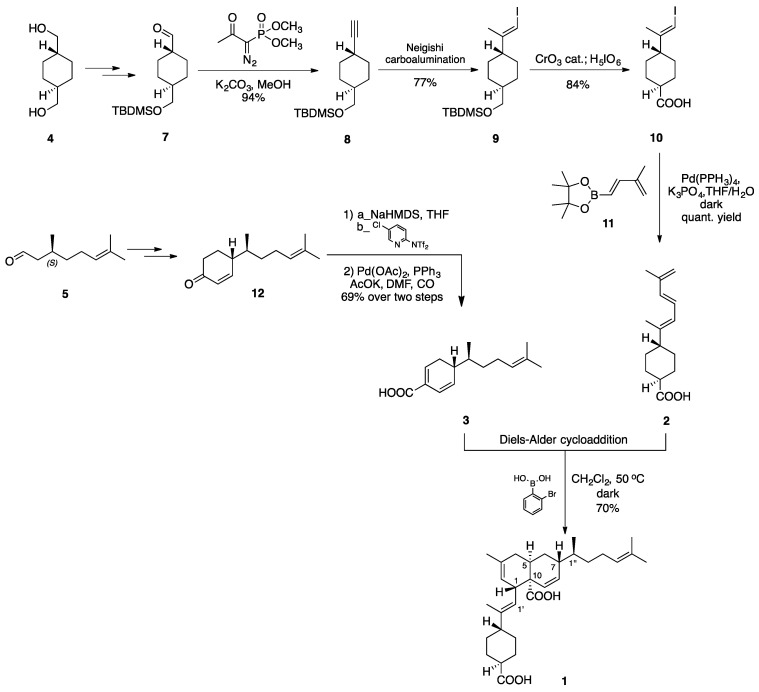
Synthesis of meiogynin A (**1**).

The biomimetic total synthesis of meiogynin A (**1**) was accomplished in 2010 by using a convergent approach allowing the determination of its absolute configuration, having an *endo* selective Diels-Alder reaction as key step [12]. The synthetic process was based on the synthesis of two synthons, the diene **2** and the dienophile **3**, obtained respectively from the commercially available *trans*-1,4-cyclohexanedimethanol (**4**) and *(S)*-citronellal (**5**). To obtain the diene **2** aldehyde **7** underwent Seyferth-Gilbert homologation using the Bestmann-Ohira [13] reagent furnishing the alkyne **8** that, under Negishi carboalumination [14] conditions, gave *E*-vinyl iodide **9** in good yield and excellent stereoselectivity. The latter was selectively oxidated by using a one-pot procedure with CrO_3_-H_5_IO_6_ [15] leading to the carboxylic acid derivative **10** finally coupled with boronic ester **11** to give the desired synthon **2**. Unfortunately, the final intermediate is unstable and light sensitive, forcing to perform the Suzuki reaction in the dark, and to purify the compound by an acid-base extraction, avoiding any type of chromatographic purification. Compound **3** was obtained starting from commercially available (S)-citronellal (**5**) that via Robinson annulation [16] furnished the cyclohexenone **12**, as substrate for the carbonilation [17] reaction. With both the key intermediates onhand, the final Diels-Alder reaction was performed using 2-bromobenzenboronic acid as organocatalyst (30 mol%) at 50 °C for 72 h [18], giving the natural product **1** in good yield (60%) and excellent chemo, regio and facial selectivity (endo/exo 91:9) (Scheme 1).

The proposed approach was flexible enough to be applied to the synthesis of non-natural analogues of meiogynin A (**1**) in order to find new and more active derivatives correlated to the parent compound [19].

### 2.2. (–)-Perezoperezone

The (–)-Perezoperezone (**13**) was isolated from the gorgonian octocoral Pseudopterogorgia rigida, a Carribean soft coral [20]. It displays a dimeric structure derived from the dimerization of two units of (-)-perezone (**14**) through an enzymatic radical mediated coupling. While bisabolane sesquiterpenoids are widely distributed both on marine and terrestrial organisms, (–)-perezoperezone (**13**) represents the first example of bisabolane dimer from corals. 

**Scheme 2:**
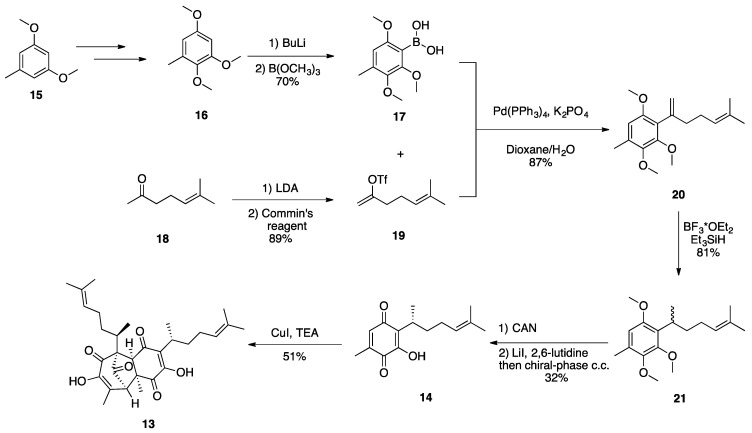
Synthesis of (–)-Perezoperezone (**13**).

The total synthesis of this marine dimer was published in 2019, and it was based on a copper-catalyzed intermolecular [5+2] homodimerization of (-)-perezone (**14**) [21]. The synthetic process started from 2,3,5-trimethoxy toluene (**16**) obtained from the commercially available 3,5-dimethoxy-toluene (**15**) via a known three-steps sequence [22]. Compound **16** was then transformed in the corresponding boronic derivative **17** after mono-lithiation and subsequent entrapment with thimethylborate. Boronic acid **17** underwent a cross coupling reaction with triflate **19** [23,24] under Suzuki-Miyaura [25] conditions furnishing compound **20** in excellent yield. The latter went through a chemoselective hydrogenation leading to the synthon **21** as racemate, while all attempts to enable an enantioselective hydrogenation were unfruitful. The key intermediate **21** was oxidated with cerium (IV) ammonium nitrate (CAN) and demethylated with LiI giving (±)-perezone. The racemate was resolved by using a chiral-phase column chromatography obtaining the enantiopure (-)-perezone (**14**). The biclyclo- [3.2.1]octadienone core of (–)-perezoperezone (**13**) was finally constructed through an intermolecular [5+2] homodimerization catalyzed by CuI, forming two new C-C bonds with the installation of four stereogenic centers in a single step (Scheme 2).

**Scheme 3:**
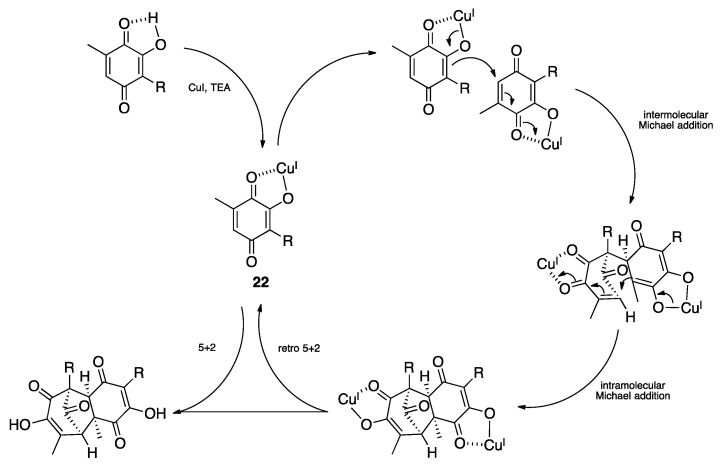
Proposed reaction mechanism for the synthesis of (–)-Perezoperezone (**13**).

The proposed mechanism proceeded through a double Michael addition thanks to the formation of the stabilized cuprate complex Cu^I^ (**22**). The presence of sterically hindered substituents (i.e., benzyl groups) on the quinone moiety induced a retro-dimerization process leaving the starting quinone untouched. A radical pathway was excluded: the yield of the dimerization process was not affected by the presence of radical scavengers such TEMPO and BHT (Scheme 3).

## 3. Xanthane Dimers

### 3.1. Mogolides A-C

The total synthesis of mogolides A-C (**24–26**) represents “a showcase of biomimetic-synthesis-guided discovery of new natural products,” wherein a natural product has been synthesized before being discovered in nature [26,27,28,29,30]. The total synthesis of these compounds, initially considered artifacts and then isolated from Xanthium mogolium Kitag plant, was accomplished in 2014 starting from xanthatin (**23**) by using photo and termal-dimerization processes [31].

**Scheme 4:**
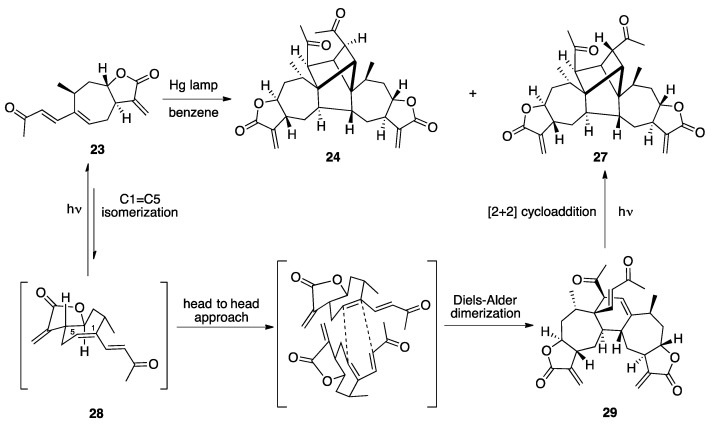
Proposed mechanism for the synthesis of mogolide A (**24**), epi-mogolide A (**27**).

The irradiation of xanthatin (**23**) with Hg lamp in benzene [32,33] provided a mixture of mogolide A (**24**) and epi-mogolide A (**27**), in a 74% combined yield, through a tandem process in which a head-to-head Diels-Alder reaction was followed by a [2+2] cycloaddition. From a mechanistic point of view, upon irradiation compound **23** could undergo C1=C5 double bond isomerization furnishing a highly reactive intermediate **28**, that quickly dimerize through a head-to-head Diels-Alder reaction giving compound **29**. The latter, after further irradiation, could transform into mogolide A (**25**) and epi-mogolide A (**27**) through an intramolecular [2+2] cycloaddition. Notably, the authors postulated the formation of intermediate **28** [34,35,36,37], characterized by an unusual trans-cycloheptenone moiety, to justify the stereochemical outcome of compound **29**. A possible radical pathway was excluded: the dimerization process proceeded smoothly in the presence of radical scavengers (Scheme 4).

**Scheme 5:**
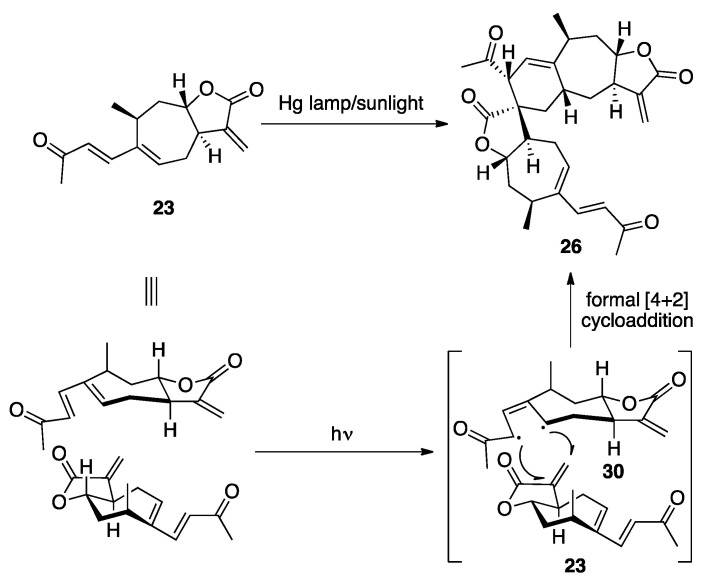
Proposed mechanism for the synthesis of mogolide C (**26**).

Mogolide C (**26**) was obtained with a different photodimerization by exposure to sunlight, or Hg lamp, xanthatin (**23**) in its solid state [38,39,40]. The final product, apparently, derived from a head-to-tail dimerization pathway where the two neighboring monomers adopted a head-to-tail stacking pattern that, under irradiation, generated a diradical intermediate **30** that reacted with a nonactivated monomer **23** through a stepwise [4+2] cycloaddition giving mogolide C (**26**). Probably the restricted molecular motion and conformation of the solid state supported the high diastereoselectivity of the reaction (Scheme 5).

**Scheme 6:**
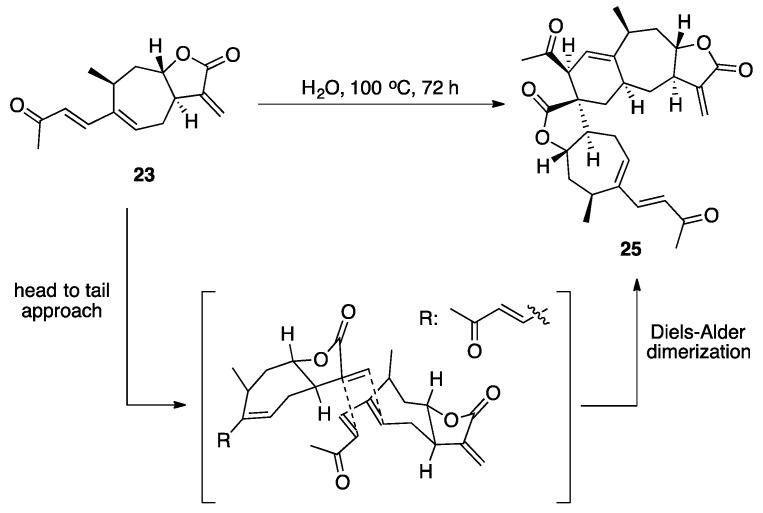
Proposed mechanism for the synthesis of mogolide B (**25**).

The last member of the magolides family, magolide B (**25**), was finally obtained by switching from a photo-induced dimerization to a thermal one. The treatment of xanthatin (**23**) in H_2_O at 100 °C for 72 h led to the formation of dimer **25** in 22% yield, through a concerted Diels-Alder reaction in a head-to-tail fashion. The reaction was successfully in H_2_O, probably due to a solvent-induced combination of hydrophobic interactions and hydrogen bonding effect (Scheme 6) [41].

### 3.2. Pungiolides A-E, L-N

Pungiolides are a group of dimeric xanthanolides isolated from different plants of genus Xanthium (Compositae) [42,43]. From a biogenetic point of view, they derive from a dimerization process of two moities of 8-epi-xanthatin (**31**) through a head-to-tail [4+2] cycloaddition. Their pharmacological profile has been explored, showing a weak cytotoxic activity [44,45] and an interesting antiprotozoal action against pathogens responsible for HAT (*Trypanosoma brucei rhodesiense*) [46].

The collective synthesis of pungiolides A-E (**32–36**), L-N (**37–39**) was reported in 2017 using pungiolide D (**35**) and pre-pungiolide (**40**) as key precursors to access to all the other dimeric congeners [47].

**Scheme 7:**
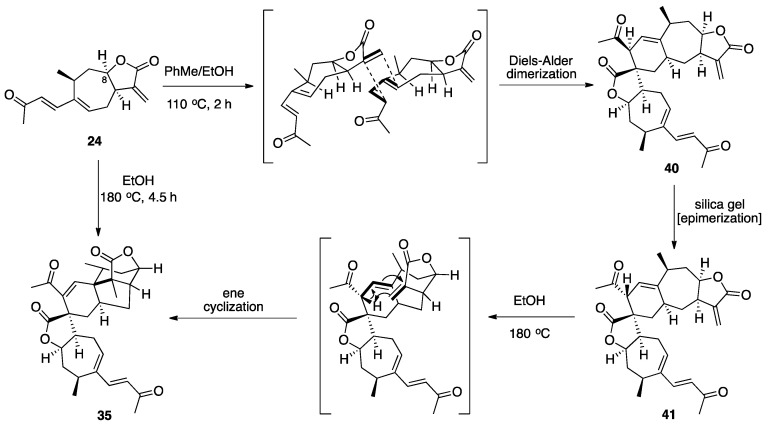
Proposed mechanism for the synthesis of pungiolide D (**35**) and pre-pungiolide (**40**).

The divergent synthesis of compounds **35** and **40** was accomplished after intensive exploration of the Diels-Alder dimerization of 8-epi-xanthatin (**31**), varying both solvents and temperature. Using a binary solvent system (PhMe/EtOH) at 110 °C, the only product obtained was pre-pungiolide (**40**) in 30% yield. Interestingly, the latter readily underwent silica-gel–induced epimerization at C-3′ giving the corresponding epi-pre-pungiolide (**41**) that at 180 °C in EtOH was converted in pungiolide D (**35**) in nearly quantitative yield, through an intramolecular ene cyclization. Alternatively, compound **35** was directly obtained in 40% yield as sole product, by performing the dimerization reaction in EtOH at 180 °C for 4.5 h (Scheme 7).

**Scheme 8:**
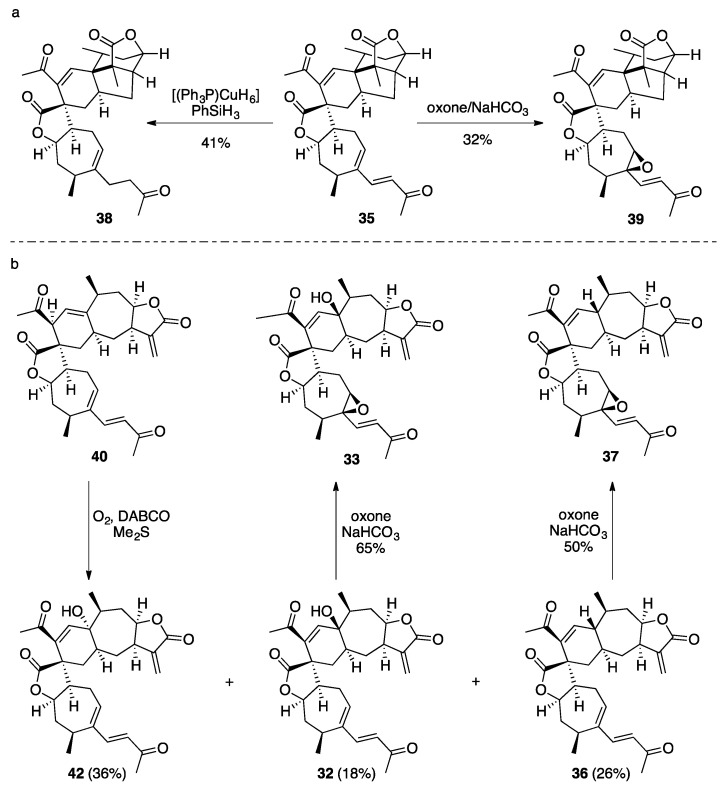
Collective total synthesis of a series of pungiolides. (**a**) M (**38**) and N (**39**); (**b**) A (**32**), B (**33**), E (**36**) and L (37).

The synthesis of the two key intermediates paved the way to obtain the other dimeric congeners through a late-stage functionalization. In this way, pungiolides M (**38**) and N (**39**) were achieved by a divergent manipulation of the C2=C3 double bond of pungiolide D (**35**): the selective reduction with Stryker reagent [(Ph_3_P)CuH]_6_ furnished compound **38** in 41% yield [48], while the epoxidation with oxone/NaHCO_3_ gave compound **39** (Scheme 8a) [49].

Pungionolide A (**32**) and its C-11 epimer (**42**) were directly obtained from pre-pungionolide (**40**), through an allylic oxidation, upon treatment with DABCO in presence of molecular oxygen followed by a reductive work-up (Me_2_S). In the same conditions, pungionolide E (**36**), a double-bond migration product of **40**, was also formed in 26% yield. Finally, **32** and **36** gave pungiolides B (**33**) and L (**37**), respectively, after regio- and diastereoselective epoxidation of C1=C5 double bond with oxone/NaHCO_3_ (Scheme 8b) [49].

## 4. Guaiane Dimers

### Ainsliadimer A, B and Gochnatiolides A-C

Ainsliadimers A and B (**43**, **44**) with gochnatiolides A–C (**45**, **46**, **47**) are members of the guaiane disesquiterpenoids dimers, showing complex and unique structures mainly characterized by an intriguing spiro [4,5] decane moiety. Ainsliadimer A and B (**43**, **44**) [50,51] have been isolated from different plants of genus Ainsliaea, while the genera Gochnatia represents the major source of gochnatiolides A–C (**45**, **46**, **47**) [43,52,53]. Whereas (+)-ainsliadimer A (**43**) displayed an interesting anti-inflammatory activity, through the inhibition of the production of NO in RAW264.7 stimulated by LPS, [54] and gochnatiolide B (**46**) demonstrated a potent anti-bladder cancer activity inducing G1 arrest both in vitro and in vivo studies, [55] the biological profile of their congeners still remains an uncharted area. Four different biomimetic total syntheses have been published for both ainsliadimers and gochnatiolides sharing dehydrozaluzanin C (**49**) [56] as key monomer, and a hetero Diels-Alder reaction as dimerization step.

**Scheme 9:**
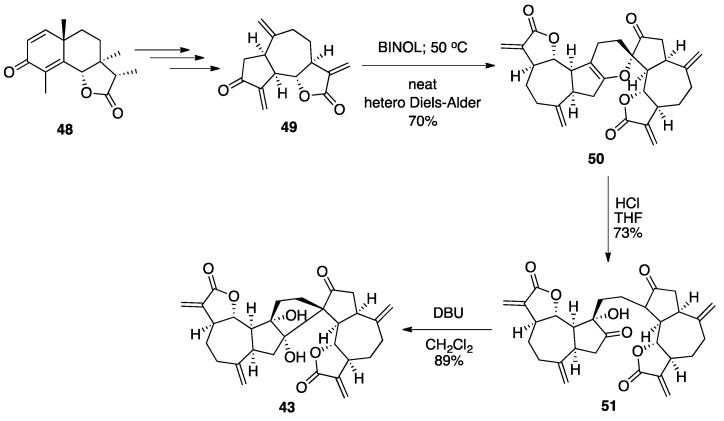
Total synthesis of (+)-ainsliadimer A (**43**).

The biomimetic total synthesis of (+)-ainsliadimer A (**43**) was reported in 2010 starting from the commercially available sesquiterpene lactone santonin (**48**) [54]. The first part of the synthetic process was dedicated to the synthesis of dehydrozaluzanin C (**49**) as key monomer, obtained in ten synthetic steps from **48**. With synthon **49** onhand, the hetero Diels-Alder dimerization was deeply studied by testing different conditions, pointing out how high temperatures and the presence of Lewis acids led to a total decomposition of the starting material. The turning point was the introduction of hydrogen bond donors as catalyst, obtaining the best results of conversion and yield with BINOL. Eventually, compound **50** was obtained in 71% yield in presence of 1 eq. of catalyst at 50 °C for 60 h without solvent. The (+)-ainsliadimer A (**43**) was finally obtained through a mild acidic hydrolysis followed by an intramolecular aldol reaction in high diluted conditions. A possible explanation for the positive action of BINOL could be its bidentate nature that helped the orientation of the two monomers allowing the [4+2] hetero-cycloaddition (Scheme 9).

**Scheme 10:**
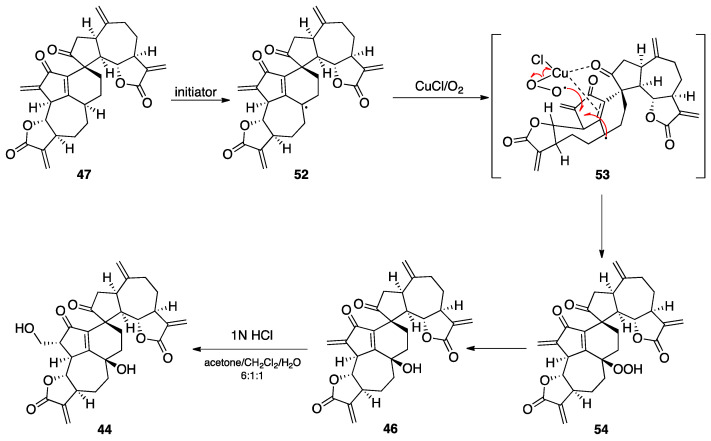
Lei’s approach to (−)-ainsliadimer B (**44**) and gochnatiolide B (**46**).

In parallel studies, two different research groups accomplished the collective total synthesis of (−)-ainsliadimer B (**44**) and gochnatiolides A-C (**45**, **46**, **47**). In the synthetic strategy proposed by Lei [57], dehydrozaluzanin C (**49**) was transformed in its silyl enol ether derivative **52**, and the two monomers went through a cross hetero Diels-Alder dimerization induced by Pd(OAc)_2_, giving a mixture of gochnatiolides A-C (**45**, **46**, **47**) in 6.7:1:1.5 ratio, respectively. The reaction was proved to be very flexible and the products ratio could be easily tuned just by varying the conditions applied and the additives added. The reaction was conducted in an anaerobic atmosphere providing only compound **47** in 14% yield, suggesting how oxygen was fundamental for the allylic oxidation; otherwise, the addition of a copper additive (CuCl) reversed the **45**:**46** ratio from 6.7:1 to 1:4.5 (Table 1). This unprecedent “copper effect” was probably due to the ability of the metal to chelate both molecular oxygen and the carbonyl moiety as well as the alkene in the transition state **53**, leading the peroxy derivative **54** that was finally reduced to **46** [58]. The (−)-ainsliadimer B (**44**) was finally obtained in 27% yield upon treatment of **46** with 1N HCl in a ternary solvent system 6:1:1 acetone/CH_2_Cl_2_/H_2_O (Scheme 10).

**Scheme 11:**
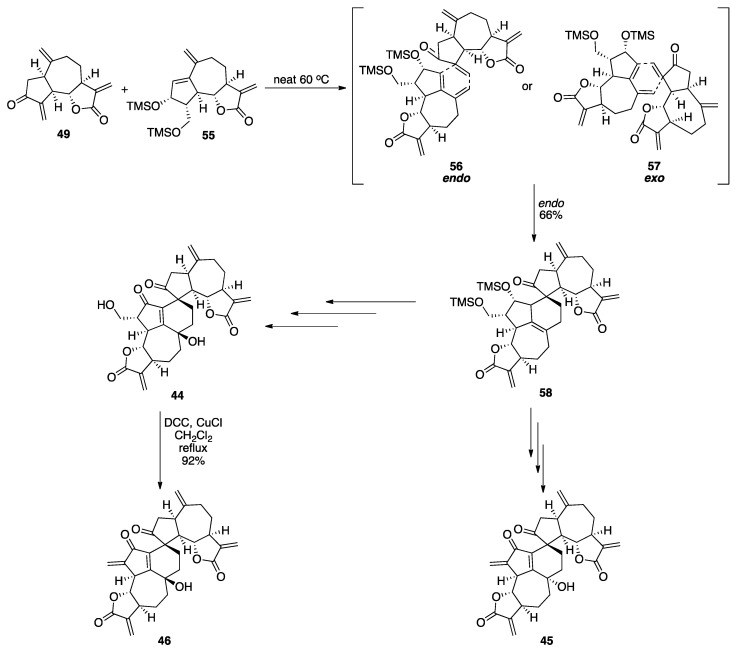
Qin’s approach to (−)-ainsliadimer B (**44**) and gochnatiolide A and B (**45**, **46**).

In the second approach, proposed by Qin [59], compound **49** was reacted with compound **55** leading as sole product the gochnatiolide-type adduct **58**, proving how the Diels-Alder cycloaddition proceeded through an *endo*-adduct transition state (**56)** rather than an *exo* one (**57**). To get the product in reasonable yield and time, the reaction had to be conducted in the solid state without solvent and additives: solvents lowered the reaction rate while the addition of any acidic additive caused the complete degradation of the starting material. With the key intermediate onhand, (−)-ainsliadimer B (**44**) and gochnatiolide A and B (**45**, **46**) were finally obtained through redox adjustments of the gochnatiolide-core **58** (Scheme 11).

**Scheme 12:**
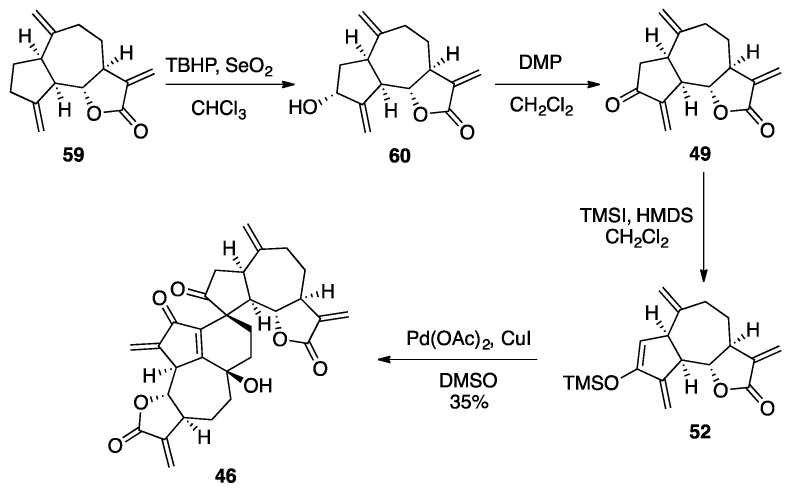
Tang’s approach to gochnatiolide B (**46**).

Recently, Tang’s group, taking advantage of the previous studies, reported a more concise synthetic strategy of gochnatiolide B (**46**) from commercially available dehydrocostunolactone (**59**) in only four synthetic steps with an overall of 26% yield. The [4+2] cycloaddition, exploiting the already described “*copper effect*,” allowed to obtain the final product in 35% yield (Scheme 12). All the reported synthetic endeavors fostered the characterization of the pharmacological profile of this natural dimer, that exhibited a potent inhibitory activity in in vivo studies against proliferation of human cancer cell lines by acting as a multi-target agent [55].

## 5. Lindenane Dimers

### 5.1. Lindenane [2+2] Dimers: Chloranthalactone F

The (+)-Chloranthalactone F (**61**) was first isolated from the leaves of Chlorantus glaber, and it is characterized by an intriguing structure with twelve stereogenic centers and two clyclopropane rings bearing two adjacent angular methyl groups next to a highly congested cyclobutane [60,61]. While the plants of the genera Chloranthus have been widely used in traditional Chinese medicine, the biological profile of this compound still remains an untouched area. Biogenetically, it is postulated that the complex architecture of chloranthalactone F (**61**) derives from an intermolecular [2+2] cycloaddition between two units of chloranthalactone A (**63**). This biogenetical hypothesis inspired two different biomimetic total synthesis.

**Scheme 13:**
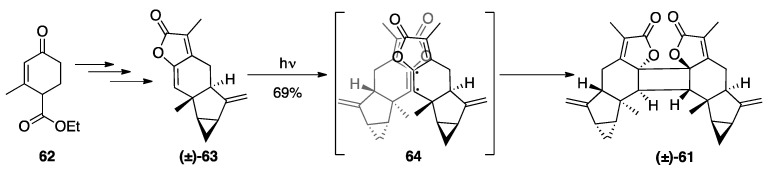
Liu’s approach to (±)-chloranthalactone F (**61**).

In the synthetic strategy proposed by Liu, (±)-chloranthalactone F (**61**) was synthetized through a photodimerization of (±)-chloranthalactone A (**63**) obtained from Hagemman’s ester (**62**) in fifteen steps [62,63]. Eventually, a hexane solution of **63** was irradiated with a Hg lamp leading the desired dimer (±)-**61** as single diastereoisomer in a yield 69%. The authors suggested that the high diastereoselectivity was given by the perfect electrical and orbital interaction between γ-alkylidenbutenolide segments in the transition state **64** (Scheme 13).

**Scheme 14:**
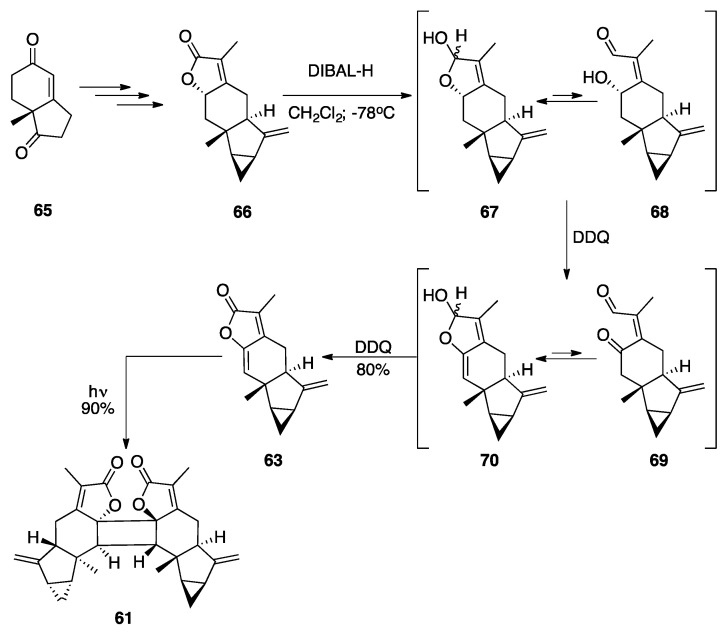
Zhao’s approach to (+)-chloranthalactone F (**61**).

In the total synthesis proposed by Zhao, enantiopure chloranthalactone A (**63**) was obtained starting from (R)-Hajos–Wiechert ketone (**65**) [64] in fourteen steps with a DDQ-involved oxidative enol-lactonization as a key step [65]. Compound **66** was first reduced with DIBAL-H furnishing the corresponding hemiacetal **67** in equilibrium with its open form **68** that, exposed to DDQ, underwent a one-pot double oxidation sequence, giving compound **63** in 80% yield. At this juncture, the exposure of a highly diluted solution (0.004 M) of the latter to a Hg lamp, furnished enantiopure (+)-chloranthalactone F (**61**) in in a yield of 92% (Scheme 14).

### 5.2. Lindenane [4+2] Dimers: Shizukaols A, C, D, E, I, Chlorajaponilide C, Multistalide B, Sarcandrolide J and Sarglabolide I

Lindenane sesquiterpene dimers deriving from a formal [4+2] cycloaddition, represent a large group of natural compounds characterized by a basic framework of eight fused rings with more than eleven stereogenic centers. Together with an intriguing structural motif, these metabolites have an interesting pharmacological profile displaying a broad range of biological activities. Among all, shizukaol E (**71**) showed a strong inhibitory effect against the most popular NNRTI-resistant HIV-1 [66], while its congener chlorajaponilide C (**72**) was revealed to be a highly selective inhibitor of chloroquine resistant strains of Plasmodium falciparum, showing IC_50_ values in the low nanomolar range comparable to the potency of artemisinin [67].

The structural and pharmacological features of this group of secondary metabolites attracted the attention of organic chemists, inducing numerous efforts to duplicate the efficiency of natural processes under laboratory conditions [68,69]. To date, only three different total syntheses have been published, two by Liu’s group and one by Peng’s group.

Liu, in 2017, published the first total synthesis of sarcandrolide J (**73**) and shizukaol D (**74**) with a Diels-Alder reaction as key dimerization step combining the dienophile **75** with the unstable diene **76** [70]. Both synthons can be obtained in eleven steps from the common precursor **77** accessible from commercially available (+)-verbenone (**78**). The treatment of **79** with benzoic acid led to the in situ formation of the unstable diene **76** that reacted with the dienophile **75** in an endo-type head-to-head [4+2] cycloaddition, furnishing the basic skeleton **80** in 83% yield. Amazingly, the furan moiety of diene **76** remained untouched, even though furan is a well-known reactive diene in Diels–Alder cycloadditions.

**Scheme 15:**
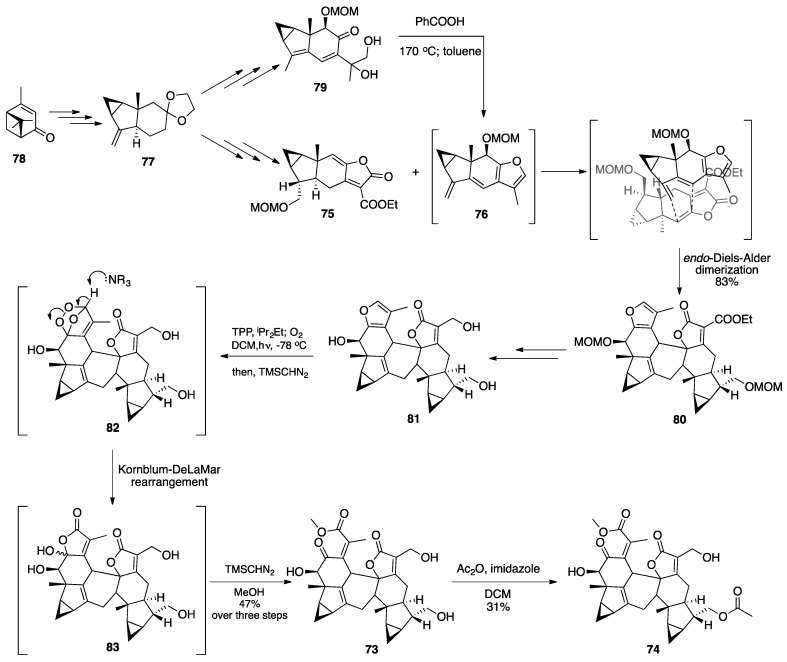
Liu’s approach to sarcandrolide J (**73**) and shizukaol D (**74**).

Manipulation of **80** furnished compound **81**, which went through a photo-oxidation reaction, giving the corresponding peroxy-derivative **82**, that after a Kornblum–DeLaMar rearrangement followed by esterification with TMSCHN_2_ produced sarcandrolide J (**74**) in 47% yield over three steps. Eventually, selective acetylation of sarcandrolide J (**73**) afforded shizukaol D (**74**) (Scheme 15).

In 2018, Peng’s group published a divergent total synthesis of shizukaol A and E (**84, 71**), involving an endo-Diels-Alder reaction between the common diene **85** and the dienophiles **63** and **86** [71]. All the three key synthons were obtained from commercially available Wieland–Miescher ketone (**87**).

**Scheme 16:**
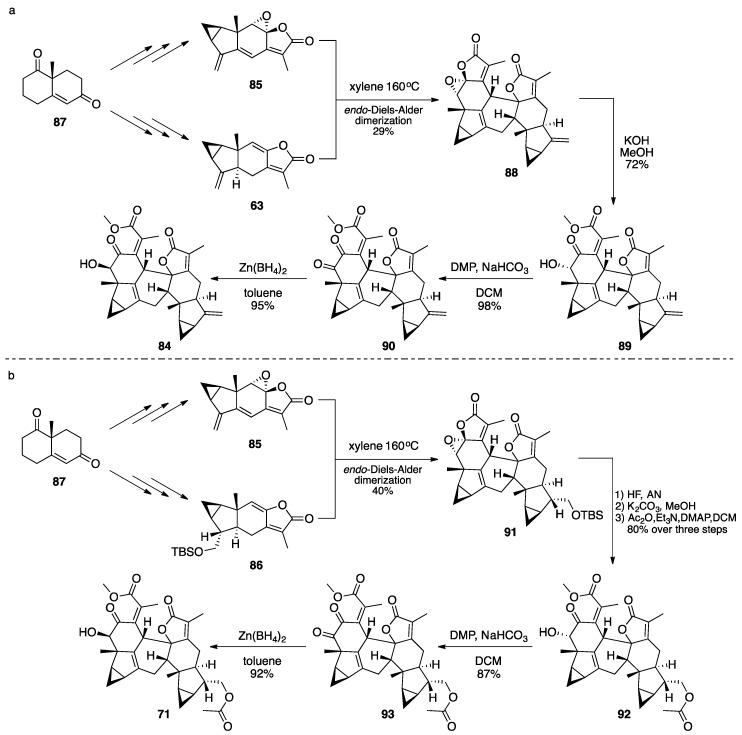
Peng’s approach to (**a**) shizukaol A (**84**), (**b**) shizukaol E (**71**).

Compound **88**, as precursor of shizukaol A (**84**), was obtained as main product by reacting the intermediate **85** with chloranthalactone A (**63**) in presence of butylated hydroxytoluene (BHT) at 160 °C in xylene. In order to “quench” the homodimerization of diene **85**, it was essential that a high diluted solution of the latter was added very slowly into a refluxing dilute solution of **63**. Alkaline and selective methanolysis of **88** furnished compound **89** that underwent epimerization of the secondary alcohol through an oxidation-reduction sequence affording the desired shizukaol A (**84**) (Scheme 16a).

Based on a similar strategy compound **91**, the basic skeleton to shizukaol E (**71**), was obtained in 40% yield by refluxing diene **85** with dienophile **86** in presence of BHT at 160 °C in xylene. Deprotection of silyl group followed by basic methanolysis and selective acetylation of primary hydroxy group furnished compound **92**, which was finally transformed in the desired shizukaol E (**71**) after epimerization of secondary alcohol (Scheme 16b).

Expanding the approach proposed by Peng, Liu in 2019 reported a divergent and unified strategy to obtain shizukaols A, C, D, I (**84**, **94**, **74**, **95**), chlorajaponilide C (**72**), multistalide B (**96**), sarcandrolide J (**73**) and sarglabolide I (**97**) by using a base-mediated thermal [4+2] cycloaddition, where the common diene **76** was reacted with dienophiles **63**, **75** and **98** [72].

**Scheme 17:**
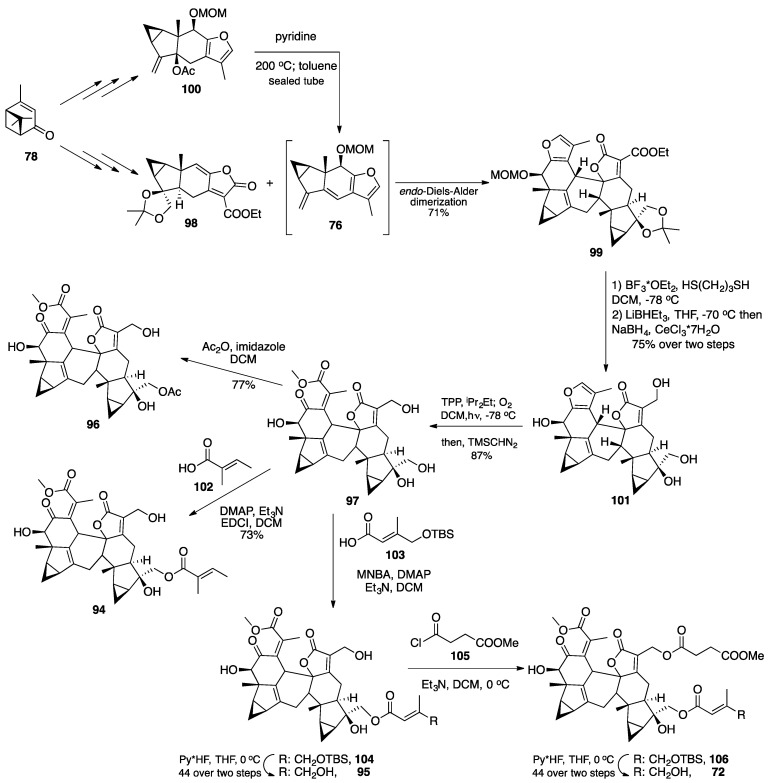
Liu’s collective synthesis to chlorajaponilide C (**72**), shizukaol C (**94**) and I (**95**), multistalide B (**96**), and sarglabolide I (**97**).

Compound **99**, the basic skeleton to chlorajaponilide C (**72**), shizukaol C (**94**) and I (**95**), multistalide B (**96**) and sarglabolide I (**97**), was obtained by reacting the diene precursor **100** with dienophile **98** in toluene and pyridine at 200 °C. The synergic action of pyridine and heating process induced the elimination of the acetate moiety from **100** generating the furyl diene **76** that underwent intermolecular [4+2] cycloaddition, giving the desired endo-adduct **99**. Global deprotection and selective reduction of the ethyl ester group furnished the intermediate **101** that was transformed in the desired sarglabolide I (**97**) through photolytic oxidation of the furan moiety, followed by methylation of the resulting acid. At this juncture, compound **97** was selectively acetylated or esterified with tiglic acid (**102**) to give respectively multistalide B (**96**) and shizukaol C (**94**). On the other side, the esterification of **97** with compound **103** gave the intermediate **104**, which after deprotection furnished shizukaol I (**95**). Chlorajaponilide C (**72**) was finally achieved through esterification of **104** with the acyl-chloride **105** followed by deprotection of the silyl group (Scheme 17).

**Scheme 18:**
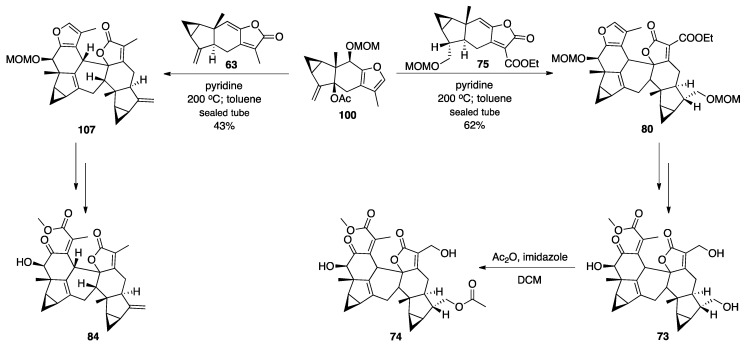
Liu’s collective synthesis to sarcandrolide J (**73**), shizukaol D (**74**) and shizukaol A (**84**).

Otherwise, the reaction between diene precursor **100** and chloranthalactone A (**63**) in toluene and pyridine at 200 °C, furnished the intermediate **107** that after acidic removal of the MOM protection and oxidative manipulation of the furan moiety furnished the desired shizukaol A (**84**) in 54% yield over two steps. The two remaining congeners were finally achieved by reacting the diene precursor **100** with the dienophile **75** leading compound **80**, that following the first generation pathway [70] led to sarcandrolide J (**73**) and shizukaol D (**74**) (Scheme 18).

## 6. Cadinane Dimers

### Gossypol

The (−)-(*R*)-Gossypol (**108**) is a polysubstituted salicylaldehyde dimer that was firstly discovered in 1886 in cotton seeds oil by Longmore [73] and purified in 1889 by cristallization from an acetic acid solution by MarchLewski [74]. Gossypol (**108**) displays different pharmacological properties and, among all, the spermicidal and antitumor activity are noteworthy. The contraceptive activity does not affect the hormone levels and derives from the inhibition of the enzyme systems that affect energy metabolism in sperm and spermatogenic cells [75]. On the other side, this polyphenolic dimer is actually on phase II clinical trials as antitumor agent against progressive or recurrent glioblastoma multiforme [76,77].

Since 1957, several groups have proposed and completed the total synthesis of this interesting natural compound, and in the last decade three different groups published their total syntheses under non-enzymatic conditions.

**Scheme 19:**
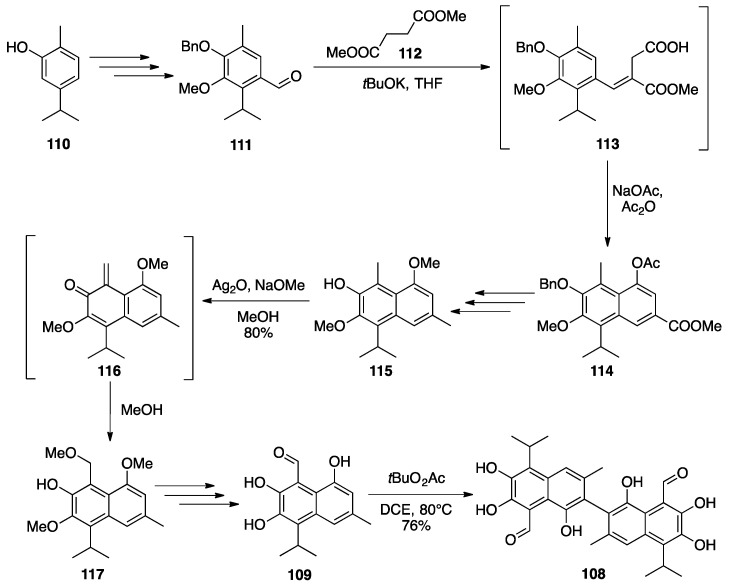
Wang’s approach to (±)-gossypol (**108**).

In 2013, Wang’s group published its approach to obtain **108** through the oxidative dimerization of two equivalents of hemigossypol (**109**) [78]. The latter was synthesized on a gram scale in eighteen steps from the commercially available carvacrol (**110**). The synthetic pathway from **110** to **109** was characterized by a Stobbe condensation between **111** and **112**, followed by an electrophilic cyclization affording the highly substituted naphthalene **114**. The latter, after protecting groups swap and redox adjustments, furnished the key intermediate **115** that underwent a Michael addition on the ortho-quinone methide **116** giving compound **117**. Finally, synthon **117** was transformed in the desired monomer **109**. At this juncture, the oxidative dimerization was achieved by treating **109** with tert-butyl peracetate (tBuO_2_Ac) in 1,2-dichloroethane at 80 °C furnishing (±)-gossypol (**108**) in 76% yield (Scheme 19).

**Scheme 20:**
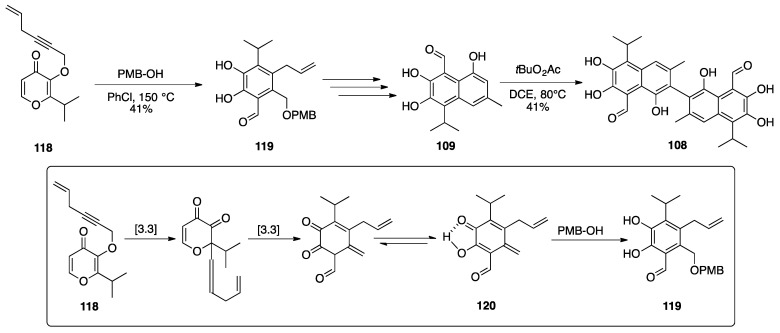
Zhu’s approach to (±)-gossypol (**108**).

In 2018, an interesting approach, based on a cascade Claisen rearrangement, was proposed by Zhu’s group where the key intermediate **119** was obtained in a single step starting from the pyrone **118** [79]. The latter was treated with *p*-methoxybenzyl alcohol in chlorobenzene at 150 °C for 13 h leading compound **119** in 41% yield with all the requested functional groups preinstalled. The authors proposed as mechanism a double allenylic-Claisen rearrangement postulating the formation of a quinone methide (**120)** easily trapped by benzylalcohol. At this juncture, compound **119** was transformed in hemigossypol **109** in fourteen steps, mainly characterized by protecting groups swap and redox adjustments. The (±)-gossypol (**108**) was obtained in 41% yield applying the same protocol previously reported by Wang (Scheme 20).

**Scheme 21:**
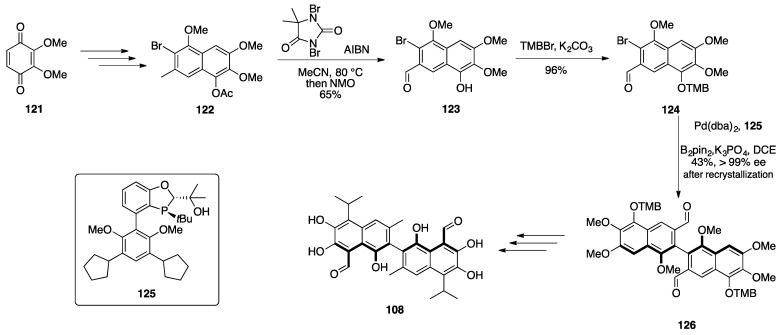
Tang’s approach to (−)-(*R*)-gossypol (**108**).

A completely different approach to obtain (−)-(*R*)-gossypol (**108**) was proposed in 2020 by Tang’s group, where an enantioselective Suzuki-Miyaura cross-coupling was used as key reaction of the synthetic pathway [80]. Enatiomerically pure (−)-**108** was obtained in ten synthetic steps from the easily accessible quinone **121** that was efficiently manipulated to obtain naphthylene **122**. A one-pot two-step sequence consisting of benzylic bromination of **122** and subsequent treatment with *N*-methylmorpholine *N*-oxide afforded the benzaldehyde **123**, that after protection of the phenol moiety as 3,4,5-trimethoxybenzyl derivative (**124**), underwent a domino Miyaura-borylation/Suzuki-coupling reaction, catalyzed by (*R*,*R*)-BaryPhos **125**, affording binaphthyl key synthon **126** in 43% yield and >99% ee after recrystallization. The latter was finally elaborated to produce (−)-(*R*)-gossypol (**108**) (Scheme 21).

## 7. Miscellaneous Dimers

### 7.1. Pinguisane Type Dimers: Bisacutifolone A and B

Bisacutifolone A and B (**127**, **128**) are two of the few examples of pinguisane-type sesquiterpenoids dimers so far identified [81,82]. They have been isolated from liverwort Porella acutifolia subsp. tosana, and despite this liverwort shown a broad range of interesting biological activities, the pharmacological profiles of the two natural dimers still remain an unexplored area. From a biogenetic point of view, it is postulated that the dimeric framework of **127** and **128** derives from an intermolecular [4+2] cycloaddition between two units of acutifolone A (**129**) [83,84]. All the reported compounds share an unusual [4.3.0]nonane framework with four contiguous chiral centers oriented in an all-*cis* fashion.

To date, two stereoselective total syntheses of bisacutifolone A and B (**127**, **128**) have been published having in common the dimerization step with two different approaches to obtain acutifolone A (**129**) as key monomer.

**Scheme 22:**
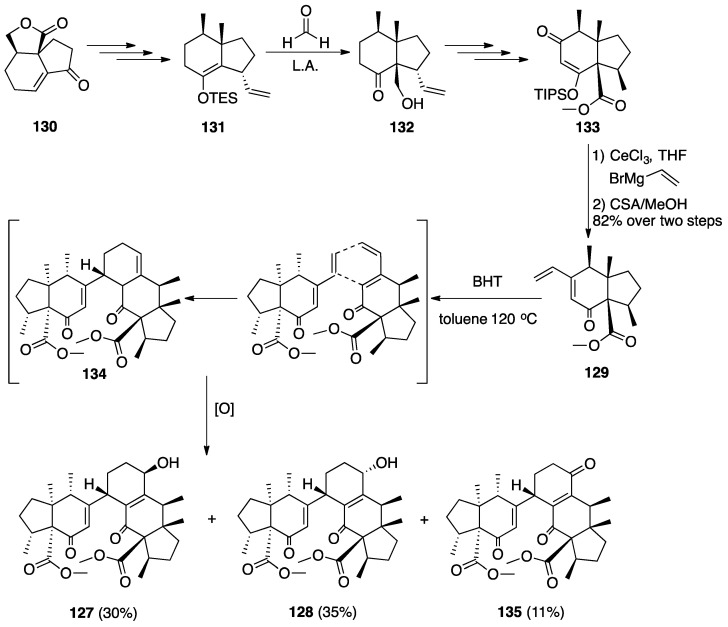
Nishiyama’s approach to bisacutifolone A (**127**) and B (**128**).

Nishiyama in 2007, published the first stereoselective total synthesis of **127** and **128** starting from the know lactone **130** [85]. The manipulation of **130** to obtain the precursor **129**, required 25 synthetic steps including a complete stereoselective Mukaiyama aldol reaction between sylilenolether **131** and formaldehyde to give compound **132**. The concentration (2.7 mmol in a 56 mL solution) and the Lewis acid used [Sc(OTf)_3_ or Yb(OTf)_3_] played a pivotal role in the success of the reaction. Having the synthon **132** onhand, redox adjustment furnished compound **133** that, after 1,2-addition of vinylmagnesium chloride in presence of CeCl_3_ followed by sylil deprotection, provided acutifolone A (**129**). Bisacutifolone A and B (**127**, **128**) were finally achieved by heating compound **129** in a sealed tube in presence of BHT in toluene at 120 °C. Computational analysis of all the possible transition states of the Diels-Alder reaction has been performed to explain the complete stereoselectivity of the dimerization step: the calculations revealed that the most stable transition state, among all the eight possible transition states, was the one furnishing compound **134**. The latter, once formed, underwent rapid auto-oxidation to give the desired dimers **127** and **128** and the overoxidized compound **135** [86,87] (Scheme 22).

**Scheme 23:**
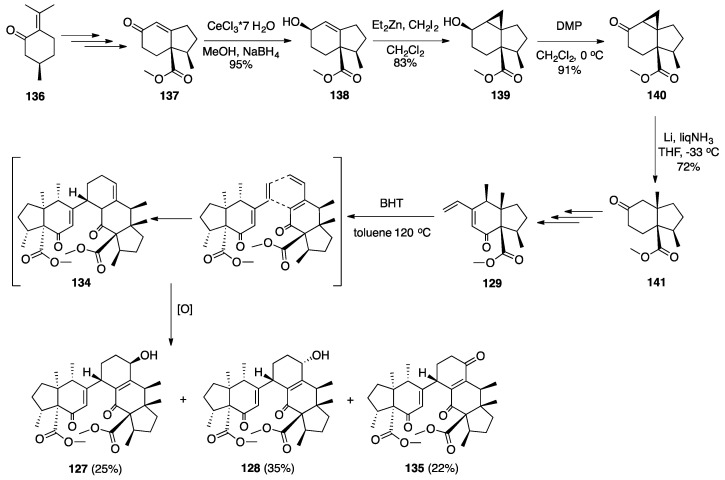
Yadav’s approach to bisacutifolone A (**127**) and B (**128**).

In 2018, Yadav published his total synthesis of bisacutifolone A (**127**) and B (**128**) starting from the bicyclic chiral synthon **137**, obtained from commercially available R-(+)-pulegone (**136**) [88]. Compound **137** was manipulated to introduce the quaternary methyl group through a four-step sequence where the allylic alcohol **138**, obtained under Luche conditions, underwent a stereospecific Furukawa’s modified Simmon-Smith reaction leading to, as sole product, the cyclopropane **139**. The latter, after oxidation with DMP, was treated with lithium in liquid ammonia leading the desired intermediate **141**. At this juncture, additional five synthetic steps were needed to obtain acutifolone (**129**). Biomimetic Diels-Alder dimerization of the **129**, under previously reported conditions [85], finally furnished the desired bisacutifolone A (**127**) and B (**128**) (Scheme 23).

### 7.2. Vannusals A and B

Vannusals A and B (**142**, **143**) are two secondary metabolites with an unusual dimeric framework, characterized by six rings and thirteen stereocenters, isolated from the tropical strains of the interstitial marine ciliate Euplotes vannus [89]. The structural assignment was elucidated by Guella, and then revised by Nicolaou after comparison of the NMR spectral data of synthetic **144** (original assigned structure) and natural vannusal B (**143**) [89,90,91]. The campaign to elucidate the true structures of the vannusals required the synthesis of eight diastereoisomers of vannusal B (**143**), in the attempt to pinpoint the correct configuration(s) of the stereocenter(s). Herein reported is the final synthetic pathway applied to obtain the desired natural dimers **142** and **143** [92].

From a retrosynthetic point of view, two key synthons have been identified: the known enantiopure vinyl iodide **145** and the racemic tricyclic fragment **146** obtained in eleven steps from the diketo intermediate **147** [90,93]. The union of these two intermediates was obtained by lithiation of **145** with t-BuLi followed by the addition of **146** furnishing a mixture of the two diastereoisomers **148** and **149** in 1:1 ratio. At this juncture, the latter was manipulated to obtain compound **150** that underwent SmI_2_-mediated cyclization leading to polycyclic product **151** as single diastereoisomer in 82% yield. Finally, compound **151** after protecting-group swap, redox adjustment and complete deprotection furnished the desired compound **143**, whose NMR and CD spectra were perfectly superimposable to that of the natural vannusal B.

**Scheme 24:**
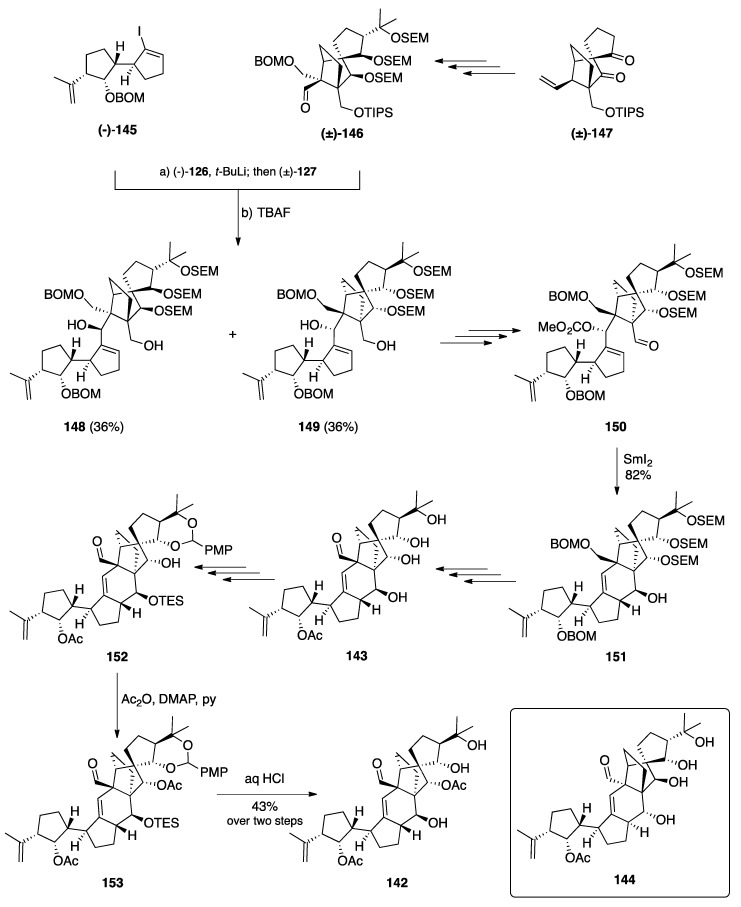
Total synthesis of vannusal A (**142**) and B (**143**).

Vannusal A (**142**) was instead obtained in four synthetic steps from the congener **143**, through acetylation of compound **152** and complete deprotection of **153** by mild acid hydrolysis. Furthermore, in this case the physical properties of **142** matched with those reported for the natural vannusal A (Scheme 24).

## 8. Sesquiterpenoid Alkaloid Dimers

### Nuphar Thioalkaloids

Dimeric nuphar thioalkaloids are a unique group of sulfur-containing secondary metabolites deriving from the dimerization of monomers possessing a regular sesquiterpenic skeleton incorporated into 3-furyl substituted piperidines. They have been isolated for the first time in 1964 from *Nuphar lutea* (L.) Sm., a yellow water lily belonging to the aquatic family of *Nymphaeaceae*, and three different series are known whose structures differ in the relative configuration at the thiaspirane junction [94]. Recently they have displayed an interesting anticancer activity with IC_50_ values in the high nanomolar range in in vitro assays against murine (B16) melanoma cells proliferation, and crude extracts of N. lutea have shown a synergistic effect enhancing the cytotoxicity of cisplatin toward Hodgkin’s lymphoma-derived cells (L428) [95,96]. Additionally, these extracts have shown an interesting anti-inflammatory activity through the inhibition of the nuclear factor kB (NFkB) [96].

**Scheme 25:**
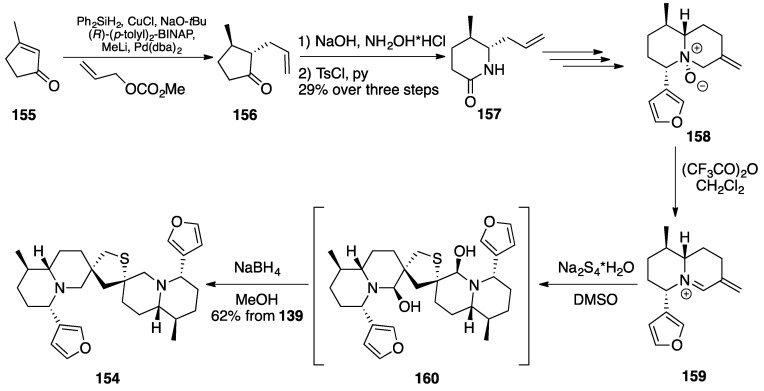
Total synthesis of (−)-neothiobinupharidine (**154**).

Although these natural compounds have been discovered in the second half of the last century, only in 2013 did Shenvi’s group report the first total synthesis of a thiaspirane nuphar dimer in enantiomerically pure form [97]. The (−)-neothiobinupharidine (**154**) was obtained in just eight synthetic steps starting from enone **155** that was selectively allylated under modified Tsuji-Trost allylation conditions, allowing to obtain compound **156** that underwent Beckmann rearrangement leading the corresponding 6-allyl piperidone **157**. The latter was readily transformed in the *trans*-quinolizidine *N*-oxide **158** that, treated with trifluoroacetic anhydride, furnished the α,β-unsaturated iminium ion **159**. The monomer **159** is stable over 24 h at room temperature, and after extensive optimization it was found that the addition of 5 eq. of sodium tetrasulfide (Na_2_S_4_) to a concentrated solution of **159** in DMSO led to the formation of (−)-6,6′-dihydroxyneothiobinupharidine (**160**) in high conversion rate and very high diastereoselectivity (10:1). Treatment of the crude mixture with NaBH_4_ allowed isolation of the desired (−)-neothiobinupharidine (**154**) in 62% yield from the *N*-oxide **158** (Scheme 25).

Expanding the approach proposed by Shenvi, Wu’s group in 2015 published a collective total synthesis of ten dimeric nuphar thioalkaloids, both natural and unnatural, using a combination of Lewis acids and chiral ligands to induce the dimerization in a stereoselective fashion [98]. Compound **161** was chosen as monomer that readily dimerized in presence of In(OTf)_3_/(3R,8S)-**162**/Na_2_S_4_ giving dimers **163**, **164**, **165** and **166** in 1.9:0.4:1:0.3 ratio, respectively. The latters, by treatment with silver nitrate in a binary solvent system acetonitrile/H_2_O, furnished (+)-6,6′-dihydroxythiobinupharidine (**167**) and (−)-6,6′-dihydroxythionuphlutine (**168**) both natural with (−)-6,6′-dihydroxyneothiobinupharidine (**160**) and 6,6′-dihydroxyneothionuphlutine (**169**) predicted natural but not yet isolated. Finally, (+)-6-hydroxythiobinupharidine (**170**) was obtained from **167** through a selective monoreduction at position C-6′ by using NaBH_4_ in cold methanol. This selective reduction was effective only on compound **167**: similar attempts on **160**, **168** and **169** resulted in complex mixtures of reduction products. The corresponding not-natural or predicted not-natural enantiomers were obtained in the same conditions by using (3S,8R)-**162**.

**Scheme 26:**
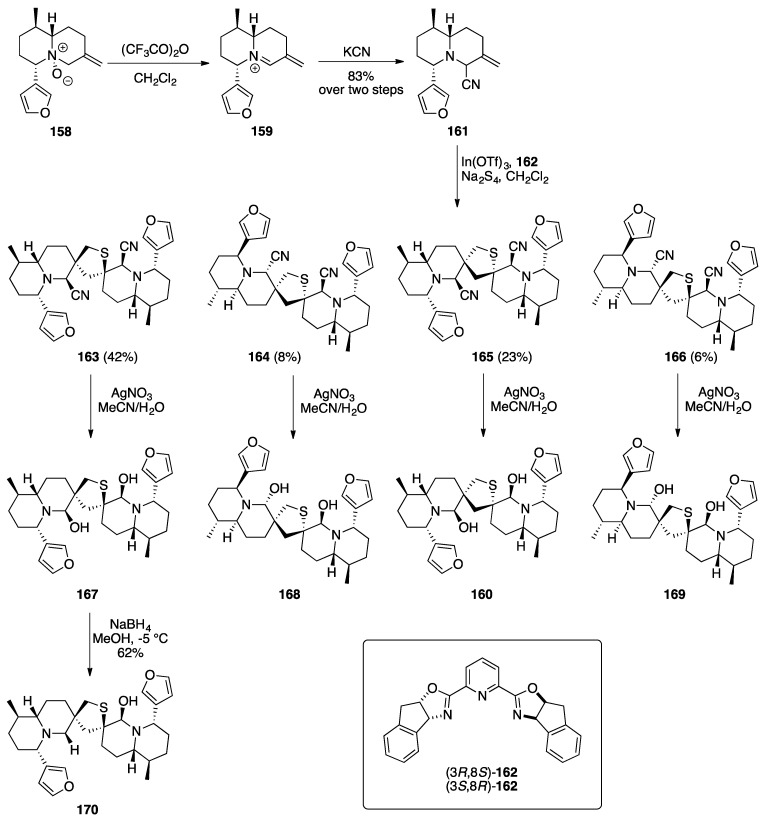
Total synthesis of (−)-6,6′-dihydroxyneothiobinupharidine (**160**), (+)-6,6′-dihydroxythiobinupharidine (**167**), (−)-6,6′-dihydroxythionuphlutine (**168**), 6,6′-dihydroxyneothionuphlutine (**169**) and (+)-6-hydroxythiobinupharidine (**170**).

All the final dimers were tested for their apoptotic properties against human U937 cell line exhibiting nearly the same activity with IC_50_ values in the low micromolar range, but among all, the predicted natural derivative **160** proved to be the most potent (Scheme 26).

**Scheme 27:**
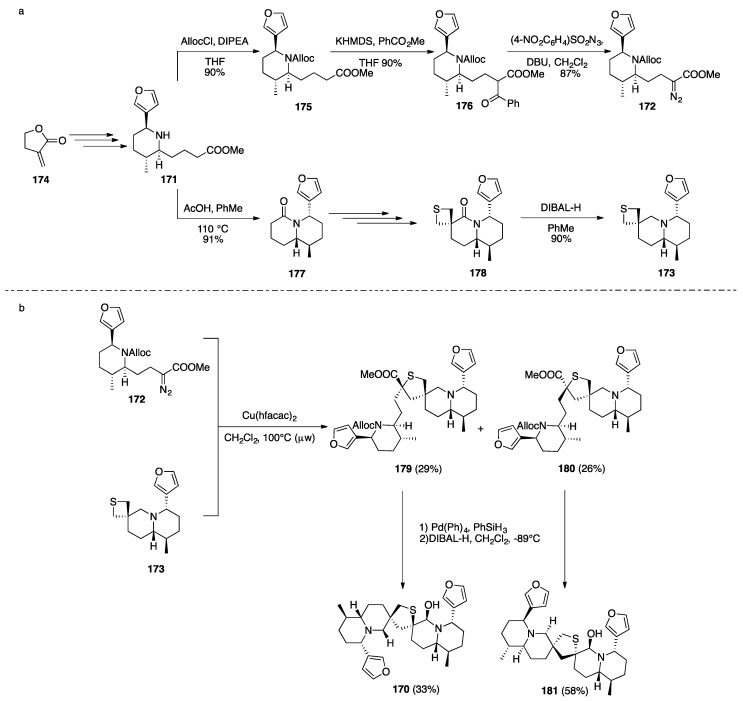
(**a**) Synthesis of coupling synthons **171** and **173**, (**b**) synthesis of (+)-6-hydroxythiobinupharidine (**170**) and (−)-6-hydroxythionuphlutine (**181**).

Despite the elegancy of the approaches proposed by both Shenvi and Wu, they have only been successful in the synthesis of symmetrically oxidized dimers with the exception of (+)-6-hydroxythiobinupharidine (**170**). Zakarian’s group in 2017 proposed a different strategy to gain access to the unsymmetrically oxidized congeners starting from compound **171** as common precursor to both the coupling partners **172** and **173** [99]. The key synthon **171** has been prepared in ten steps from the commercially available 2-methylene-γ-butyrolactone **174**. The first coupling partner **172** was obtained in three steps from **171** with protection of the amino group as *N*-allyloxycarbonyl derivative (**175**), followed by acylation with methyl benzoate and subsequent treatment with 4-nitrobenzenesulfonyl azide in presence of DBU to insert the diazo moiety. Otherwise, lactamization of **171** gave the bicyclic derivative **177** that, after a series of manipulations, furnished compound **178** with the thietane ring installed. The latter was finally reduced to the second requested coupling partner **173** with DIBAL-H (Scheme 27a).

Having the two key intermediates onhand, the coupling reaction was accomplished using a combination of Cu(hfacac)_2_ and microwave irradiation furnishing two epimers **179** and **180** in 29% and 26% yield, respectively. To complete the synthesis, **179** and **180** went through the removal of allyloxycarbonyl groups followed by the reduction with DIBAL-H at −89 °C, affording the unsymmetrically oxidized hemiaminals (+)-6-hydroxythiobinupharidine (**170**) and (−)-6-hyroxythiobinupharidine (**181**) (Scheme 27b).

## 9. Merosesquiterpenoid Dimers

### Indolesesquiterpenoid Dimers: Dixiamycin B and Dixiamycin C

Indole sesquiterpenoids are a small group of secondary metabolites characterized by a dichotomous structure with an indole moiety fused to a terpenic unit [100]. While different monomeric members have been isolated from Streptomyces species, displaying interesting biological activities such as anti-HIV and antibiotic [101,102,103], dimeric congeners still remain a mere curiosity given the small number of derivatives identified. Among them, while the two atropoisomeric dixiamycin A and B (**182**, **183**) present a N-N linkage acting as connector, dixiamycin C (**184**) is characterized by C6-N1′ bond linking the two monomeric units [104,105].

**Scheme 28:**
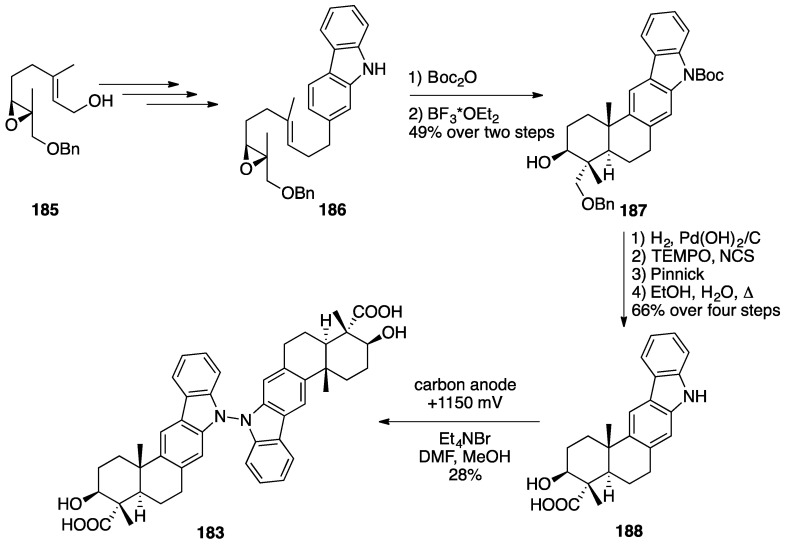
Total synthesis of dixiamycin B (**183**).

The total synthesis of dixiamycin B (**183**) was published in 2014 by Baran’s group and it was based on an elegant electrochemical dimerization process with the selective formation of the N-N linkage [106]. The synthetic pathway started from the enantioenriched epoxy-alcohol **185** readily transformed into the carbazole intermediate **186** that, after N-protection as BOC-derivative and treatment with BF_3_· OEt_2_, resulted in the desired pentacycle **187**. The latter, after complete deprotection and oxidation of the primary alcohol, furnished compound **188** on a gram scale. The desired dimer **183** was finally obtained in 28% yield by treatment of **188** in a binary solvent system (MeOH/DMF) in presence of Et_4_NBr with a carbon electrode (anode) and an applied potential of +1550 mV. At this stage, it was unclear to the authors if dixiamycin A (**182**) was formed under the reaction conditions and rapidly decomposed or the reaction was selective versus the formation of dixiamycin B (**164**) as the only dimeric product (Scheme 28).

**Scheme 29:**
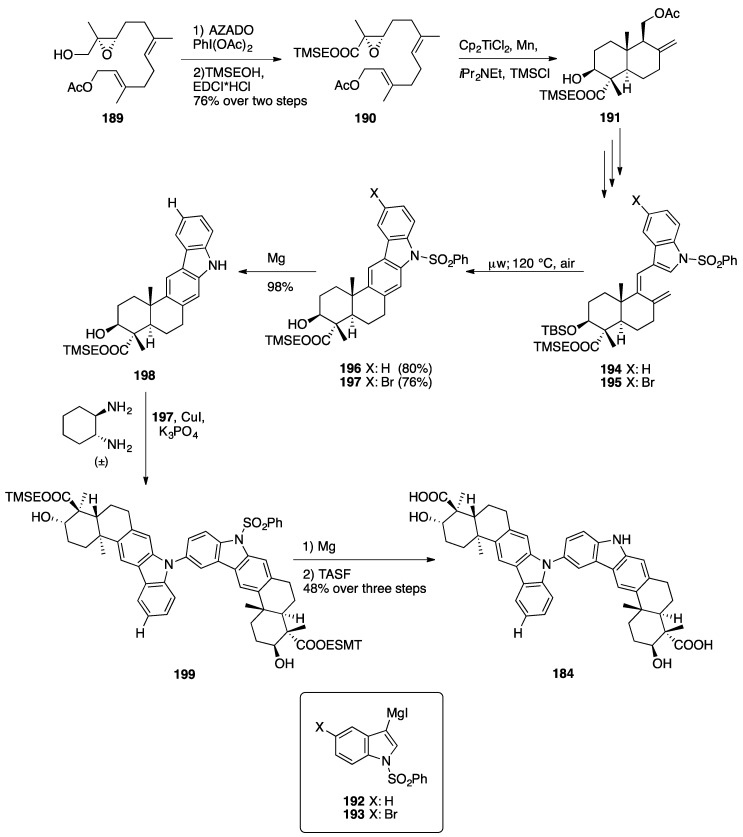
Total synthesis of dixiamycin C (**184**).

In 2015, Li and coworkers published the total synthesis of dixiamycin C (**184**) based on a thermal 6π-electrocyclization for the construction of the monomeric pentacyclic framework and a Buchwald-Hartwig [107] coupling in the dimerization step [108]. The synthetic strategy started from the optically active epoxide **189** that, after oxidative manipulation of the primary alcohol and subsequent protection of the carboxylic moiety, was readily cyclized furnishing the trans-decaline **191** as single diastereoisomer. Compound **191** passed through a sequence of protecting-group swap, redox adjustments, Grignard (**192** or **193**) addition and final dehydration, giving compound **194** and its brominated analogue **195**. The trienes **194** and **195** under microwave irradiation underwent thermal 6π-electrocyclization and a subsequent aromatization to generate the terpene-carbazole analogues **196** and **197**. At this juncture, desulfonylation of **196** and subsequent coupling with **197** under Buchwald’s protocol [109], furnished **199** that finally, after complete deprotection, afforded the desired dixiamycin C (**184**) (Scheme 29).

## 10. Conclusions

Bioactive natural compounds are the products of continuous adaptation of living organisms to a constantly changing environment, magnifying the ability of nature in the construction of complex architectures. Dimeric sesquiterpenoids are an excellent example of this adaptation, being characterized by unique and complex frameworks combined with a wide range of biological activities. In the last decade, more than 400 disesquiterpenoids have been isolated, showing more potent activities than their corresponding monomeric congeneres. Despite their interesting pharmacological profile, further studies are blocked or slowed down by the difficulties in obtaining these secondary metabolites in good amount and purity.

The attempt to duplicate the efficiency and the selectivity of natural processes under laboratory conditions still remains a challenging problem in organic chemistry, stimulating the development of new synthetic methodologies to build complex frameworks in a shorter time and more efficient manner. This review has summarized all the latest (2010–2020) efforts devoted to the synthesis of disequiterpenoids using new biomimetic approaches that allowed the access to interesting secondary metabolites otherwise produced by nature in very small amounts.

Although many strides have been made, there is still a lot of work to be done: only a very small number of disesquiterpenoids have been synthetized and characterized by a pharmacological standpoint. New and more efficient synthetic pathways will pave the way for a further and deep exploration of the biological profile of this relatively untouched group of secondary metabolites, with the possibility to qualify some of them as possible candidates to be used as a foundation and starting point in novel drug discovery processes.

## Figures and Tables

**Table 1:** Biomimetic synthesis of gochnatiolides.

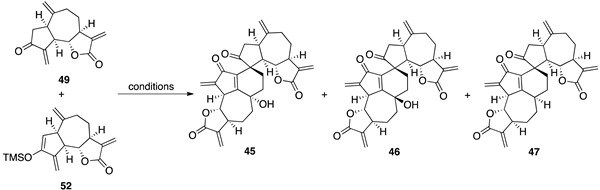			
**Conditions**	**45 ^a^**	**46 ^a^**	**47 ^a^**
Pd(OAc)_2_/DMSO/air/50 °C	40	6	9
Pd(OAc)_2_/DMSO/glovebox/50 °C	0	0	14
Pd(OAc)_2_/DMSO/CuCl/air/50 °C	6	27	5

^a^ Isolated yields.

## Data Availability

Not applicable.

## Protecting Groups Abbreviations

TBDMS (*tert*-butyldimethylsilyl); TMS (trimethylsilyl); TES (triethylsilyl); TMSE (2-(trimethylsilyl)ethyl), TIPS (triisopropylsilyl); SEM (2-(Trimethylsilyl)ethoxymethyl); MOM (methoxymethyl); BOM (Benzyl Chloromethyl Ether); PMB (*p*-methoxybenzyl).
